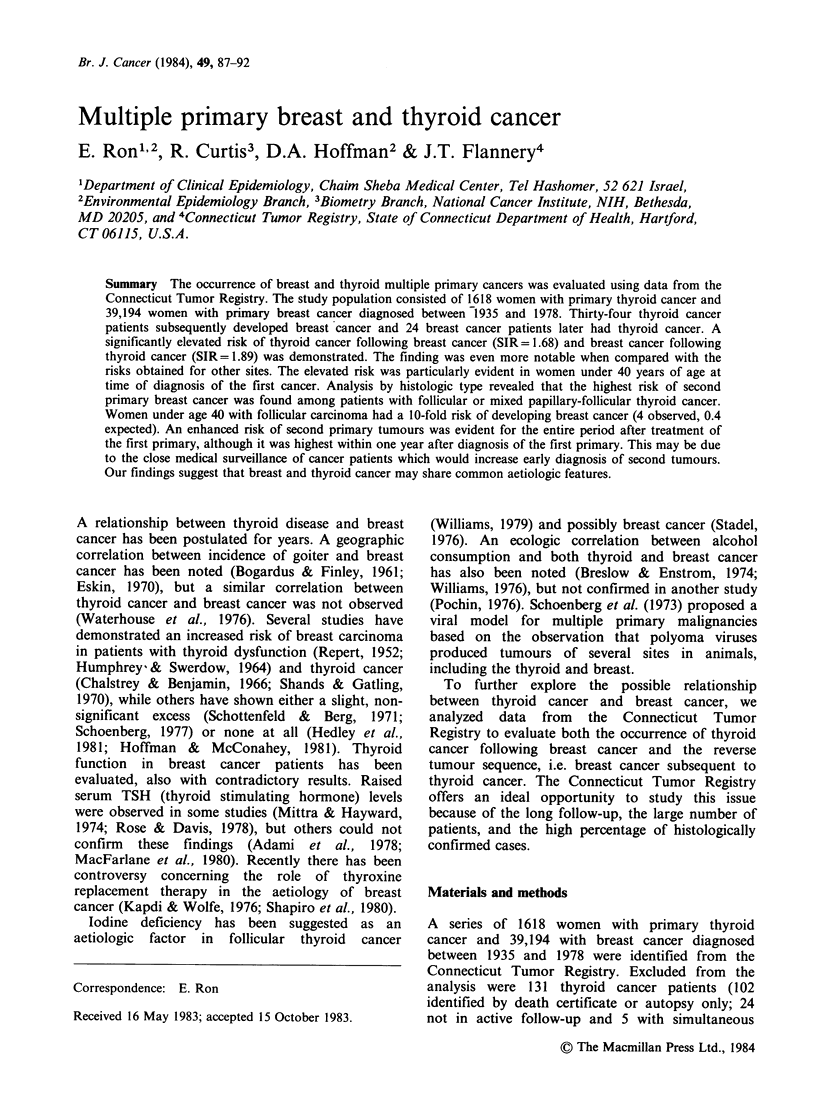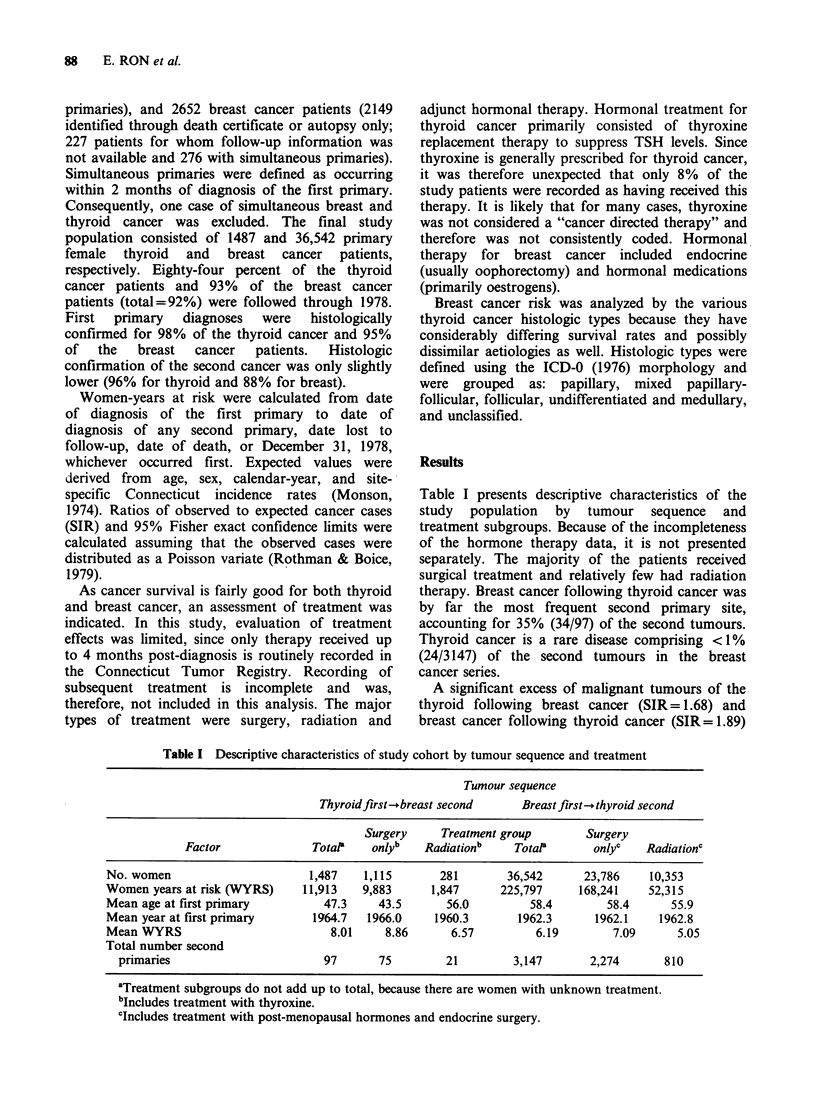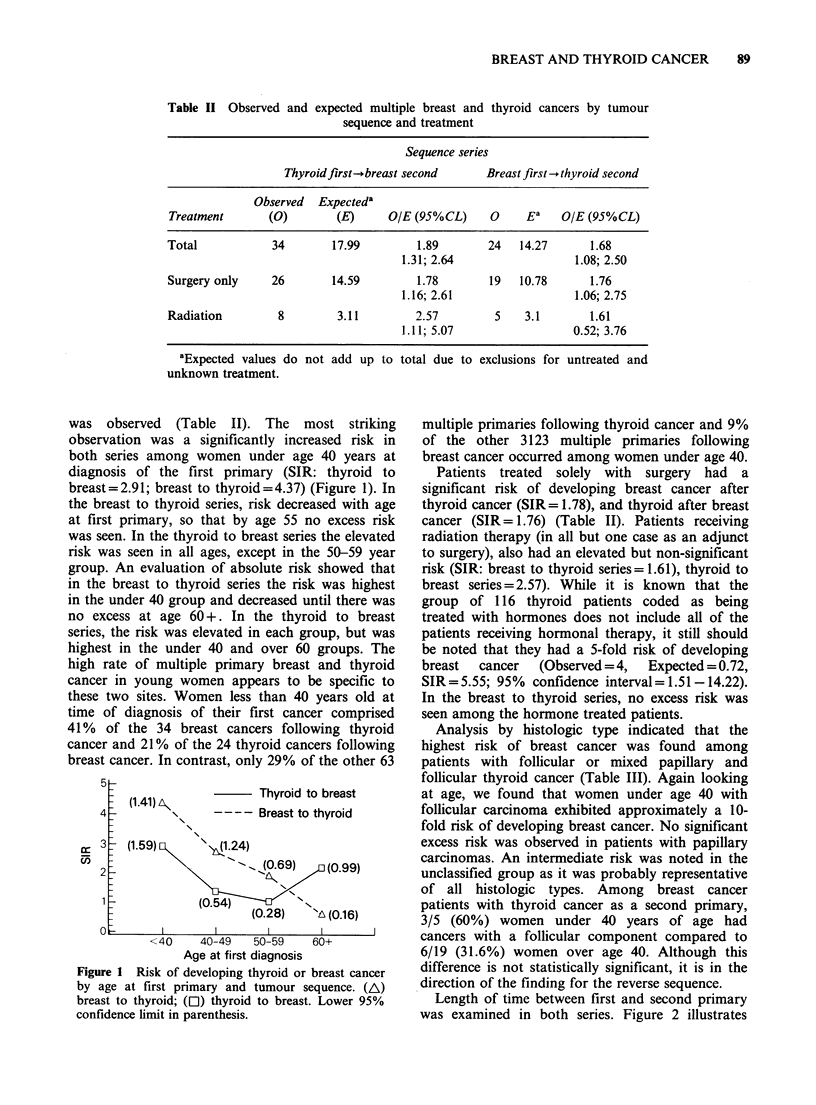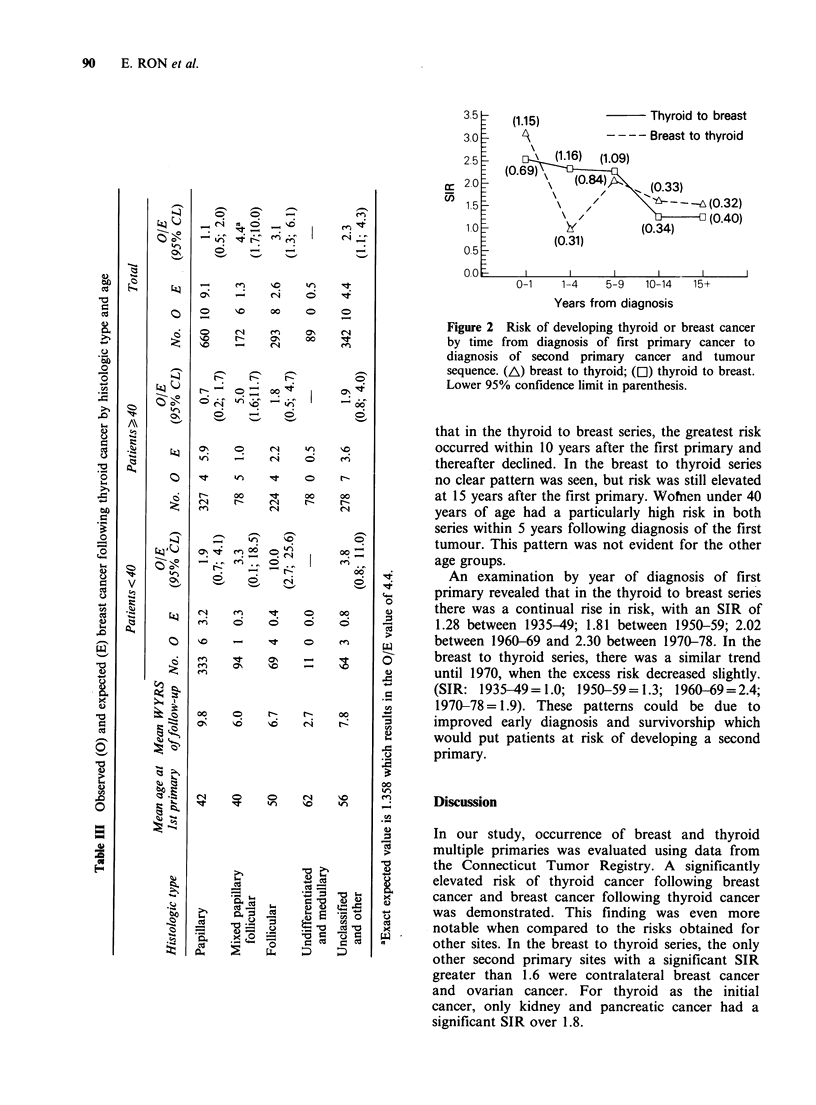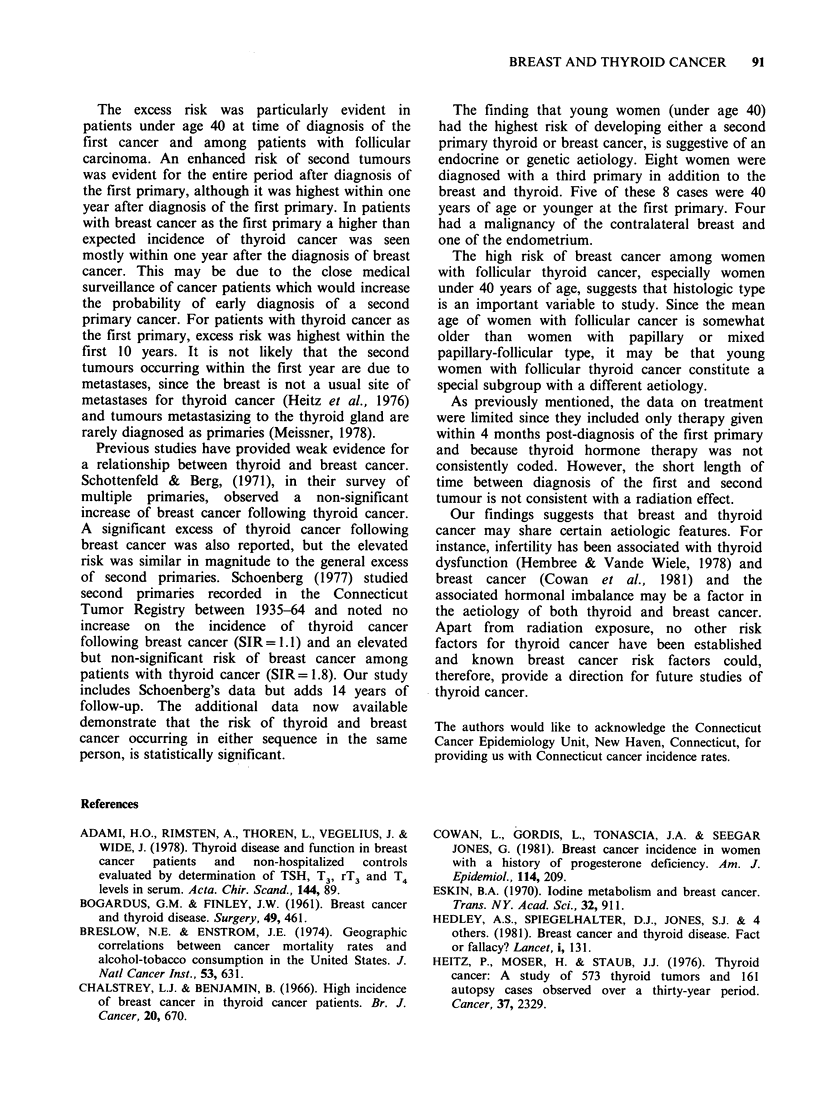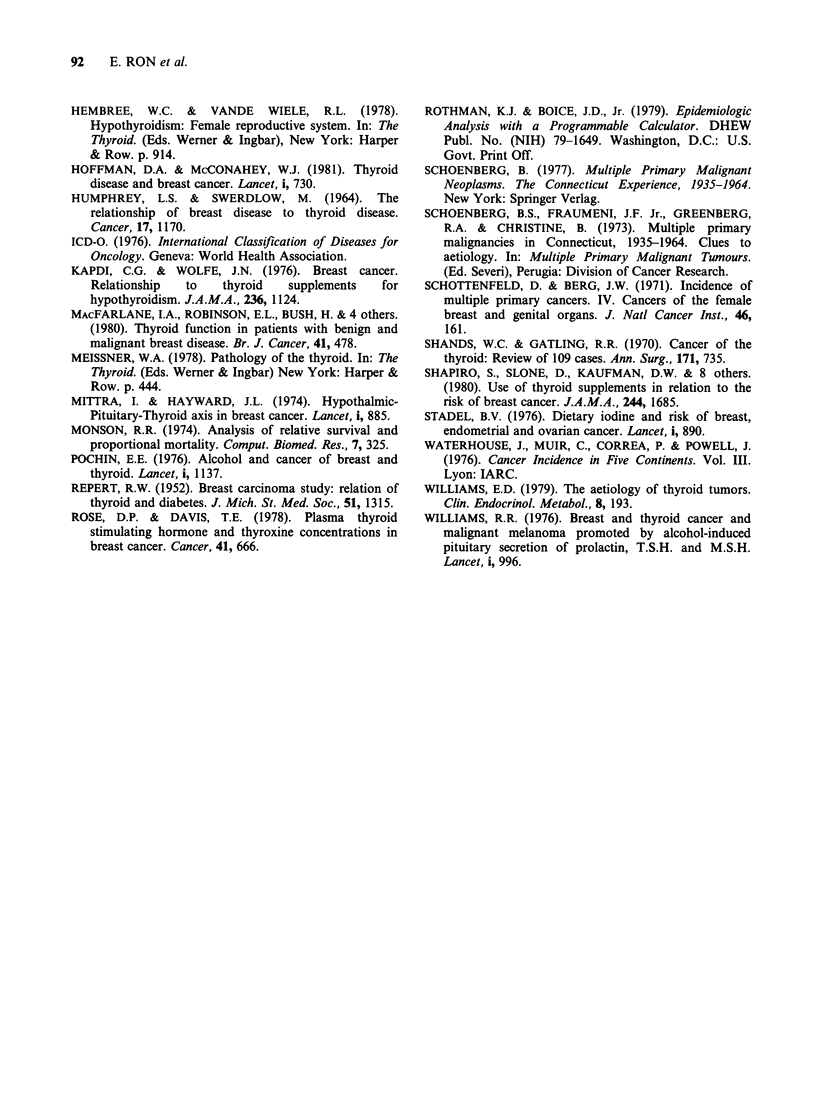# Multiple primary breast and thyroid cancer.

**DOI:** 10.1038/bjc.1984.13

**Published:** 1984-01

**Authors:** E. Ron, R. Curtis, D. A. Hoffman, J. T. Flannery

## Abstract

The occurrence of breast and thyroid multiple primary cancers was evaluated using data from the Connecticut Tumor Registry. The study population consisted of 1618 women with primary thyroid cancer and 39,194 women with primary breast cancer diagnosed between 1935 and 1978. Thirty-four thyroid cancer patients subsequently developed breast cancer and 24 breast cancer patients later had thyroid cancer. A significantly elevated risk of thyroid cancer following breast cancer (SIR = 1.68) and breast cancer following thyroid cancer (SIR = 1.89) was demonstrated. The finding was even more notable when compared with the risks obtained for other sites. The elevated risk was particularly evident in women under 40 years of age at time of diagnosis of the first cancer. Analysis by histologic type revealed that the highest risk of second primary breast cancer was found among patients with follicular or mixed papillary-follicular thyroid cancer. Women under age 40 with follicular carcinoma had a 10-fold risk of developing breast cancer (4 observed, 0.4 expected). An enhanced risk of second primary tumours was evident for the entire period after treatment of the first primary, although it was highest within one year after diagnosis of the first primary. This may be due to the close medical surveillance of cancer patients which would increase early diagnosis of second tumours. Our findings suggest that breast and thyroid cancer may share common aetiologic features.


Br. J. Cancer (1984), 49, 87–92

### Multiple primary breast and thyroid cancer

E. Ron[1,2], R. Curtis[3], D.A. Hoffman[2] & J.T. Flannery[4]

[1]*Department of Clinical Epidemiology, Chaim Sheba Medical Center, Tel Hashomer, 52 621 Israel,* [2]*Environmental Epidemiology Branch,* [3]*Biometry Branch, National Cancer Institute, NIH, Bethesda, MD 20205, and* [4]*Connecticut Tumor Registry, State of Connecticut Department of Health, Hartford, CT 06115, U.S.A.*

**Summary** The occurrence of breast and thyroid multiple primary cancers was evaluated using data from the Connecticut Tumor Registry. The study population consisted of 1618 women with primary thyroid cancer and 39,194 women with primary breast cancer diagnosed between 1935 and 1978. Thirty-four thyroid cancer patients subsequently developed breast cancer and 24 breast cancer patients later had thyroid cancer. A significantly elevated risk of thyroid cancer following breast cancer (SIR = 1.68) and breast cancer following thyroid cancer (SIR = 1.89) was demonstrated. The finding was even more notable when compared with the risks obtained for other sites. The elevated risk was particularly evident in women under 40 years of age at time of diagnosis of the first cancer. Analysis by histologic type revealed that the highest risk of second primary breast cancer was found among patients with follicular or mixed papillary-follicular thyroid cancer. Women under age 40 with follicular carcinoma had a 10-fold risk of developing breast cancer (4 observed, 0.4 expected). An enhanced risk of second primary tumours was evident for the entire period after treatment of the first primary, although it was highest within one year after diagnosis of the first primary. This may be due to the close medical surveillance of cancer patients which would increase early diagnosis of second tumours. Our findings suggest that breast and thyroid cancer may share common aetiologic features.

A relationship between thyroid disease and breast cancer has been postulated for years. A geographic correlation between incidence of goiter and breast cancer has been noted (Bogardus & Finley, 1961; Eskin, 1970), but a similar correlation between thyroid cancer and breast cancer was not observed (Waterhouse *et al.*, 1976). Several studies have demonstrated an increased risk of breast carcinoma in patients with thyroid dysfunction (Repert, 1952; Humphrey & Swerdow, 1964) and thyroid cancer (Chalstrey & Benjamin, 1966; Shands & Gatling, 1970), while others have shown either a slight, non-significant excess (Schottenfeld & Berg, 1971; Schoenberg, 1977) or none at all (Hedley *et al.*, 1981; Hoffman & McConahey, 1981). Thyroid function in breast cancer patients has been evaluated, also with contradictory results. Raised serum TSH (thyroid stimulating hormone) levels were observed in some studies (Mittra & Hayward, 1974; Rose & Davis, 1978), but others could not confirm these findings (Adami *et al.*, 1978; MacFarlane *et al.*, 1980). Recently there has been controversy concerning the role of thyroxine replacement therapy in the aetiology of breast cancer (Kapdi & Wolfe, 1976; Shapiro *et al.*, 1980).

Iodine deficiency has been suggested as an aetiologic factor in follicular thyroid cancer (Williams, 1979) and possibly breast cancer (Stadel, 1976). An ecologic correlation between alcohol consumption and both thyroid and breast cancer has also been noted (Breslow & Enstrom, 1974; Williams, 1976), but not confirmed in another study (Pochin, 1976). Schoenberg *et al.* (1973) proposed a viral model for multiple primary malignancies based on the observation that polyoma viruses produced tumours of several sites in animals, including the thyroid and breast.

To further explore the possible relationship between thyroid cancer and breast cancer, we analyzed data from the Connecticut Tumor Registry to evaluate both the occurrence of thyroid cancer following breast cancer and the reverse tumour sequence, i.e. breast cancer subsequent to thyroid cancer. The Connecticut Tumor Registry offers an ideal opportunity to study this issue because of the long follow-up, the large number of patients, and the high percentage of histologically confirmed cases.

### Materials and methods

A series of 1618 women with primary thyroid cancer and 39,194 with breast cancer diagnosed between 1935 and 1978 were identified from the Connecticut Tumor Registry. Excluded from the analysis were 131 thyroid cancer patients (102 identified by death certificate or autopsy only; 24 not in active follow-up and 5 with simultaneous

Correspondence: E. Ron

Received 16 May 1983; accepted 15 October 1983.

© The Macmillan Press Ltd., 1984

primaries), and 2652 breast cancer patients (2149 identified through death certificate or autopsy only; 227 patients for whom follow-up information was not available and 276 with simultaneous primaries). Simultaneous primaries were defined as occurring within 2 months of diagnosis of the first primary. Consequently, one case of simultaneous breast and thyroid cancer was excluded. The final study population consisted of 1487 and 36,542 primary female thyroid and breast cancer patients, respectively. Eighty-four percent of the thyroid cancer patients and 93% of the breast cancer patients (total = 92%) were followed through 1978. First primary diagnoses were histologically confirmed for 98% of the thyroid cancer and 95% of the breast cancer patients. Histologic confirmation of the second cancer was only slightly lower (96% for thyroid and 88% for breast).

Women-years at risk were calculated from date of diagnosis of the first primary to date of diagnosis of any second primary, date lost to follow-up, date of death, or December 31, 1978, whichever occurred first. Expected values were derived from age, sex, calendar-year, and site-specific Connecticut incidence rates (Monson, 1974). Ratios of observed to expected cancer cases (SIR) and 95% Fisher exact confidence limits were calculated assuming that the observed cases were distributed as a Poisson variate (Rothman & Boice, 1979).

As cancer survival is fairly good for both thyroid and breast cancer, an assessment of treatment was indicated. In this study, evaluation of treatment effects was limited, since only therapy received up to 4 months post-diagnosis is routinely recorded in the Connecticut Tumor Registry. Recording of subsequent treatment is incomplete and was, therefore, not included in this analysis. The major types of treatment were surgery, radiation and adjunct hormonal therapy. Hormonal treatment for thyroid cancer primarily consisted of thyroxine replacement therapy to suppress TSH levels. Since thyroxine is generally prescribed for thyroid cancer, it was therefore unexpected that only 8% of the study patients were recorded as having received this therapy. It is likely that for many cases, thyroxine was not considered a "cancer directed therapy" and therefore was not consistently coded. Hormonal therapy for breast cancer included endocrine (usually oophorectomy) and hormonal medications (primarily oestrogens).

Breast cancer risk was analyzed by the various thyroid cancer histologic types because they have considerably differing survival rates and possibly dissimilar aetiologies as well. Histologic types were defined using the ICD-0 (1976) morphology and were grouped as: papillary, mixed papillary-follicular, follicular, undifferentiated and medullary, and unclassified.

### Results

Table I presents descriptive characteristics of the study population by tumour sequence and treatment subgroups. Because of the incompleteness of the hormone therapy data, it is not presented separately. The majority of the patients received surgical treatment and relatively few had radiation therapy. Breast cancer following thyroid cancer was by far the most frequent second primary site, accounting for 35% (34/97) of the second tumours. Thyroid cancer is a rare disease comprising <1% (24/3147) of the second tumours in the breast cancer series.

A significant excess of malignant tumours of the thyroid following breast cancer (SIR = 1.68) and breast cancer following thyroid cancer (SIR = 1.89)

**Table I** Descriptive characteristics of study cohort by tumour sequence and treatment

| | *Tumour sequence* | | | | | |
|---|---|---|---|---|---|---|
| | *Thyroid first→breast second* | | | *Breast first→thyroid second* | | |
| | *Treatment group* | | | | | |
| *Factor* | *Total*[a] | *Surgery only*[b] | *Radiation*[b] | *Total*[a] | *Surgery only*[c] | *Radiation*[c] |
| No. women | 1,487 | 1,115 | 281 | 36,542 | 23,786 | 10,353 |
| Women years at risk (WYRS) | 11,913 | 9,883 | 1,847 | 225,797 | 168,241 | 52,315 |
| Mean age at first primary | 47.3 | 43.5 | 56.0 | 58.4 | 58.4 | 55.9 |
| Mean year at first primary | 1964.7 | 1966.0 | 1960.3 | 1962.3 | 1962.1 | 1962.8 |
| Mean WYRS | 8.01 | 8.86 | 6.57 | 6.19 | 7.09 | 5.05 |
| Total number second primaries | 97 | 75 | 21 | 3,147 | 2,274 | 810 |

[a]Treatment subgroups do not add up to total, because there are women with unknown treatment.
[b]Includes treatment with thyroxine.
[c]Includes treatment with post-menopausal hormones and endocrine surgery.

**Table II** Observed and expected multiple breast and thyroid cancers by tumour sequence and treatment

| | *Sequence series* | | | | | |
|---|---|---|---|---|---|---|
| | *Thyroid first→breast second* | | | *Breast first→thyroid second* | | |
| *Treatment* | *Observed (O)* | *Expected[a] (E)* | *O/E (95%CL)* | *O* | *E[a]* | *O/E (95%CL)* |
| Total | 34 | 17.99 | 1.89<br>1.31; 2.64 | 24 | 14.27 | 1.68<br>1.08; 2.50 |
| Surgery only | 26 | 14.59 | 1.78<br>1.16; 2.61 | 19 | 10.78 | 1.76<br>1.06; 2.75 |
| Radiation | 8 | 3.11 | 2.57<br>1.11; 5.07 | 5 | 3.1 | 1.61<br>0.52; 3.76 |

[a]Expected values do not add up to total due to exclusions for untreated and unknown treatment.

was observed (Table II). The most striking observation was a significantly increased risk in both series among women under age 40 years at diagnosis of the first primary (SIR: thyroid to breast = 2.91; breast to thyroid = 4.37) (Figure 1). In the breast to thyroid series, risk decreased with age at first primary, so that by age 55 no excess risk was seen. In the thyroid to breast series the elevated risk was seen in all ages, except in the 50–59 year group. An evaluation of absolute risk showed that in the breast to thyroid series the risk was highest in the under 40 group and decreased until there was no excess at age 60+. In the thyroid to breast series, the risk was elevated in each group, but was highest in the under 40 and over 60 groups. The high rate of multiple primary breast and thyroid cancer in young women appears to be specific to these two sites. Women less than 40 years old at time of diagnosis of their first cancer comprised 41% of the 34 breast cancers following thyroid cancer and 21% of the 24 thyroid cancers following breast cancer. In contrast, only 29% of the other 63

**Figure 1** Risk of developing thyroid or breast cancer by age at first primary and tumour sequence. (△) breast to thyroid; (□) thyroid to breast. Lower 95% confidence limit in parenthesis.

multiple primaries following thyroid cancer and 9% of the other 3123 multiple primaries following breast cancer occurred among women under age 40.

Patients treated solely with surgery had a significant risk of developing breast cancer after thyroid cancer (SIR = 1.78), and thyroid after breast cancer (SIR = 1.76) (Table II). Patients receiving radiation therapy (in all but one case as an adjunct to surgery), also had an elevated but non-significant risk (SIR: breast to thyroid series = 1.61), thyroid to breast series = 2.57). While it is known that the group of 116 thyroid patients coded as being treated with hormones does not include all of the patients receiving hormonal therapy, it still should be noted that they had a 5-fold risk of developing breast cancer (Observed = 4, Expected = 0.72, SIR = 5.55; 95% confidence interval = 1.51 – 14.22). In the breast to thyroid series, no excess risk was seen among the hormone treated patients.

Analysis by histologic type indicated that the highest risk of breast cancer was found among patients with follicular or mixed papillary and follicular thyroid cancer (Table III). Again looking at age, we found that women under age 40 with follicular carcinoma exhibited approximately a 10-fold risk of developing breast cancer. No significant excess risk was observed in patients with papillary carcinomas. An intermediate risk was noted in the unclassified group as it was probably representative of all histologic types. Among breast cancer patients with thyroid cancer as a second primary, 3/5 (60%) women under 40 years of age had cancers with a follicular component compared to 6/19 (31.6%) women over age 40. Although this difference is not statistically significant, it is in the direction of the finding for the reverse sequence.

Length of time between first and second primary was examined in both series. Figure 2 illustrates

**Table III** Observed (O) and expected (E) breast cancer following thyroid cancer by histologic type and age

| *Histologic type* | *Mean age at 1st primary* | *Mean WYRS of follow-up* | *Patients<40* *No.* | *O* | *E* | *O/E (95% CL)* | *Patients≥40* *No.* | *O* | *E* | *O/E (95% CL)* | *Total* *No.* | *O* | *E* | *O/E (95% CL)* |
|---|---|---|---|---|---|---|---|---|---|---|---|---|---|---|
| Papillary | 42 | 9.8 | 333 | 6 | 3.2 | 1.9 (0.7; 4.1) | 327 | 4 | 5.9 | 0.7 (0.2; 1.7) | 660 | 10 | 9.1 | 1.1 (0.5; 2.0) |
| Mixed papillary follicular | 40 | 6.0 | 94 | 1 | 0.3 | 3.3 (0.1; 18.5) | 78 | 5 | 1.0 | 5.0 (1.6;11.7) | 172 | 6 | 1.3 | 4.4[a] (1.7;10.0) |
| Follicular | 50 | 6.7 | 69 | 4 | 0.4 | 10.0 (2.7; 25.6) | 224 | 4 | 2.2 | 1.8 (0.5; 4.7) | 293 | 8 | 2.6 | 3.1 (1.3; 6.1) |
| Undifferentiated and medullary | 62 | 2.7 | 11 | 0 | 0.0 | — | 78 | 0 | 0.5 | — | 89 | 0 | 0.5 | — |
| Unclassified and other | 56 | 7.8 | 64 | 3 | 0.8 | 3.8 (0.8; 11.0) | 278 | 7 | 3.6 | 1.9 (0.8; 4.0) | 342 | 10 | 4.4 | 2.3 (1.1; 4.3) |

[a]Exact expected value is 1.358 which results in the O/E value of 4.4.

**Figure 2** Risk of developing thyroid or breast cancer by time from diagnosis of first primary cancer to diagnosis of second primary cancer and tumour sequence. (△) breast to thyroid; (□) thyroid to breast. Lower 95% confidence limit in parenthesis.

that in the thyroid to breast series, the greatest risk occurred within 10 years after the first primary and thereafter declined. In the breast to thyroid series no clear pattern was seen, but risk was still elevated at 15 years after the first primary. Women under 40 years of age had a particularly high risk in both series within 5 years following diagnosis of the first tumour. This pattern was not evident for the other age groups.

An examination by year of diagnosis of first primary revealed that in the thyroid to breast series there was a continual rise in risk, with an SIR of 1.28 between 1935–49; 1.81 between 1950–59; 2.02 between 1960–69 and 2.30 between 1970–78. In the breast to thyroid series, there was a similar trend until 1970, when the excess risk decreased slightly. (SIR: 1935–49 = 1.0; 1950–59 = 1.3; 1960–69 = 2.4; 1970–78 = 1.9). These patterns could be due to improved early diagnosis and survivorship which would put patients at risk of developing a second primary.

### Discussion

In our study, occurrence of breast and thyroid multiple primaries was evaluated using data from the Connecticut Tumor Registry. A significantly elevated risk of thyroid cancer following breast cancer and breast cancer following thyroid cancer was demonstrated. This finding was even more notable when compared to the risks obtained for other sites. In the breast to thyroid series, the only other second primary sites with a significant SIR greater than 1.6 were contralateral breast cancer and ovarian cancer. For thyroid as the initial cancer, only kidney and pancreatic cancer had a significant SIR over 1.8.

The excess risk was particularly evident in patients under age 40 at time of diagnosis of the first cancer and among patients with follicular carcinoma. An enhanced risk of second tumours was evident for the entire period after diagnosis of the first primary, although it was highest within one year after diagnosis of the first primary. In patients with breast cancer as the first primary a higher than expected incidence of thyroid cancer was seen mostly within one year after the diagnosis of breast cancer. This may be due to the close medical surveillance of cancer patients which would increase the probability of early diagnosis of a second primary cancer. For patients with thyroid cancer as the first primary, excess risk was highest within the first 10 years. It is not likely that the second tumours occurring within the first year are due to metastases, since the breast is not a usual site of metastases for thyroid cancer (Heitz *et al.*, 1976) and tumours metastasizing to the thyroid gland are rarely diagnosed as primaries (Meissner, 1978).

Previous studies have provided weak evidence for a relationship between thyroid and breast cancer. Schottenfeld & Berg, (1971), in their survey of multiple primaries, observed a non-significant increase of breast cancer following thyroid cancer. A significant excess of thyroid cancer following breast cancer was also reported, but the elevated risk was similar in magnitude to the general excess of second primaries. Schoenberg (1977) studied second primaries recorded in the Connecticut Tumor Registry between 1935–64 and noted no increase on the incidence of thyroid cancer following breast cancer (SIR = 1.1) and an elevated but non-significant risk of breast cancer among patients with thyroid cancer (SIR = 1.8). Our study includes Schoenberg's data but adds 14 years of follow-up. The additional data now available demonstrate that the risk of thyroid and breast cancer occurring in either sequence in the same person, is statistically significant.

The finding that young women (under age 40) had the highest risk of developing either a second primary thyroid or breast cancer, is suggestive of an endocrine or genetic aetiology. Eight women were diagnosed with a third primary in addition to the breast and thyroid. Five of these 8 cases were 40 years of age or younger at the first primary. Four had a malignancy of the contralateral breast and one of the endometrium.

The high risk of breast cancer among women with follicular thyroid cancer, especially women under 40 years of age, suggests that histologic type is an important variable to study. Since the mean age of women with follicular cancer is somewhat older than women with papillary or mixed papillary-follicular type, it may be that young women with follicular thyroid cancer constitute a special subgroup with a different aetiology.

As previously mentioned, the data on treatment were limited since they included only therapy given within 4 months post-diagnosis of the first primary and because thyroid hormone therapy was not consistently coded. However, the short length of time between diagnosis of the first and second tumour is not consistent with a radiation effect.

Our findings suggests that breast and thyroid cancer may share certain aetiologic features. For instance, infertility has been associated with thyroid dysfunction (Hembree & Vande Wiele, 1978) and breast cancer (Cowan *et al.*, 1981) and the associated hormonal imbalance may be a factor in the aetiology of both thyroid and breast cancer. Apart from radiation exposure, no other risk factors for thyroid cancer have been established and known breast cancer risk factors could, therefore, provide a direction for future studies of thyroid cancer.

The authors would like to acknowledge the Connecticut Cancer Epidemiology Unit, New Haven, Connecticut, for providing us with Connecticut cancer incidence rates.

### References

ADAMI, H.O., RIMSTEN, A., THOREN, L., VEGELIUS, J. & WIDE, J. (1978). Thyroid disease and function in breast cancer patients and non-hospitalized controls evaluated by determination of TSH, $T_3$, $rT_3$ and $T_4$ levels in serum. *Acta. Chir. Scand.*, **144**, 89.

BOGARDUS, G.M. & FINLEY, J.W. (1961). Breast cancer and thyroid disease. *Surgery*, **49**, 461.

BRESLOW, N.E. & ENSTROM, J.E. (1974). Geographic correlations between cancer mortality rates and alcohol-tobacco consumption in the United States. *J. Natl Cancer Inst.*, **53**, 631.

CHALSTREY, L.J. & BENJAMIN, B. (1966). High incidence of breast cancer in thyroid cancer patients. *Br. J. Cancer*, **20**, 670.

COWAN, L., GORDIS, L., TONASCIA, J.A. & SEEGAR JONES, G. (1981). Breast cancer incidence in women with a history of progesterone deficiency. *Am. J. Epidemiol.*, **114**, 209.

ESKIN, B.A. (1970). Iodine metabolism and breast cancer. *Trans. NY. Acad. Sci.*, **32**, 911.

HEDLEY, A.S., SPIEGELHALTER, D.J., JONES, S.J. & 4 others. (1981). Breast cancer and thyroid disease. Fact or fallacy? *Lancet*, **i**, 131.

HEITZ, P., MOSER, H. & STAUB, J.J. (1976). Thyroid cancer: A study of 573 thyroid tumors and 161 autopsy cases observed over a thirty-year period. *Cancer*, **37**, 2329.

HEMBREE, W.C. & VANDE WIELE, R.L. (1978). Hypothyroidism: Female reproductive system. In: *The Thyroid.* (Eds. Werner & Ingbar), New York: Harper & Row. p. 914.

HOFFMAN, D.A. & MCCONAHEY, W.J. (1981). Thyroid disease and breast cancer. *Lancet*, **i**, 730.

HUMPHREY, L.S. & SWERDLOW, M. (1964). The relationship of breast disease to thyroid disease. *Cancer*, **17**, 1170.

ICD-O. (1976). *International Classification of Diseases for Oncology*. Geneva: World Health Association.

KAPDI, C.G. & WOLFE, J.N. (1976). Breast cancer. Relationship to thyroid supplements for hypothyroidism. *J.A.M.A.*, **236**, 1124.

MACFARLANE, I.A., ROBINSON, E.L., BUSH, H. & 4 others. (1980). Thyroid function in patients with benign and malignant breast disease. *Br. J. Cancer*, **41**, 478.

MEISSNER, W.A. (1978). Pathology of the thyroid. In: *The Thyroid.* (Eds. Werner & Ingbar) New York: Harper & Row. p. 444.

MITTRA, I. & HAYWARD, J.L. (1974). Hypothalmic-Pituitary-Thyroid axis in breast cancer. *Lancet*, **i**, 885.

MONSON, R.R. (1974). Analysis of relative survival and proportional mortality. *Comput. Biomed. Res.*, **7**, 325.

POCHIN, E.E. (1976). Alcohol and cancer of breast and thyroid. *Lancet*, **i**, 1137.

REPERT, R.W. (1952). Breast carcinoma study: relation of thyroid and diabetes. *J. Mich. St. Med. Soc.*, **51**, 1315.

ROSE, D.P. & DAVIS, T.E. (1978). Plasma thyroid stimulating hormone and thyroxine concentrations in breast cancer. *Cancer*, **41**, 666.

ROTHMAN, K.J. & BOICE, J.D., Jr. (1979). *Epidemiologic Analysis with a Programmable Calculator*. DHEW Publ. No. (NIH) 79–1649. Washington, D.C.: U.S. Govt. Print Off.

SCHOENBERG, B. (1977). *Multiple Primary Malignant Neoplasms. The Connecticut Experience, 1935–1964.* New York: Springer Verlag.

SCHOENBERG, B.S., FRAUMENI, J.F. Jr., GREENBERG, R.A. & CHRISTINE, B. (1973). Multiple primary malignancies in Connecticut, 1935–1964. Clues to aetiology. In: *Multiple Primary Malignant Tumours.* (Ed. Severi), Perugia: Division of Cancer Research.

SCHOTTENFELD, D. & BERG, J.W. (1971). Incidence of multiple primary cancers. IV. Cancers of the female breast and genital organs. *J. Natl Cancer Inst.*, **46**, 161.

SHANDS, W.C. & GATLING, R.R. (1970). Cancer of the thyroid: Review of 109 cases. *Ann. Surg.*, **171**, 735.

SHAPIRO, S., SLONE, D., KAUFMAN, D.W. & 8 others. (1980). Use of thyroid supplements in relation to the risk of breast cancer. *J.A.M.A.*, **244**, 1685.

STADEL, B.V. (1976). Dietary iodine and risk of breast, endometrial and ovarian cancer. *Lancet*, **i**, 890.

WATERHOUSE, J., MUIR, C., CORREA, P. & POWELL, J. (1976). *Cancer Incidence in Five Continents.* Vol. III. Lyon: IARC.

WILLIAMS, E.D. (1979). The aetiology of thyroid tumors. *Clin. Endocrinol. Metabol.*, **8**, 193.

WILLIAMS, R.R. (1976). Breast and thyroid cancer and malignant melanoma promoted by alcohol-induced pituitary secretion of prolactin, T.S.H. and M.S.H. *Lancet*, **i**, 996.